# Supplementary material for: Impacts of sediment derived from erosion of partially-constructed road on aquatic organisms in a tropical river: The Río San Juan, Nicaragua and Costa Rica
Source: PLoS One. 2020 Nov 17;15(11):e0242356. doi: 10.1371/journal.pone.0242356 (PMC7671519; doi:10.1371/journal.pone.0242356)
Supplement: S1 Table — South-bank tributary deltas designated as S-1, S-2, etc, north-bank deltas as N-1, N-2, etc. (DOCX) [file pone.0242356.s001.docx]

**S1 Table.** **Coordinates of sampled deltas along the Río San Juan, Nicaragua.** South-bank tributary deltas designated as S-1, S-2, etc, North-bank deltas as N-1, N-2, etc.

| Site | Latitude | Longitude |
| --- | --- | --- |
| *South Bank* |  |  |
| S-1 | -84.35933333300 | 10.99698500000 |
| S-2 | -84.35409933930 | 10.99030940540 |
| S-3 | -84.28382000000 | 10.89443000000 |
| S-4 | -84.28213166700 | 10.89327333300 |
| S-5 | -84.27767348230 | 10.89269348540 |
| S-6 | -84.27846253600 | 10.89264772500 |
| S-7 | -84.26815310670 | 10.89182263050 |
| S-8 | -84.26354020910 | 10.89096424330 |
| *North Bank* |  |  |
| N-1 | -84.29281034980 | 10.91394448280 |
| N-2 | -84.28700359230 | 10.90482145620 |
| N-3 | -84.28559759790 | 10.90077234720 |
| N-4 | -84.26302965570 | 10.89231645490 |
| N-5 | -84.24867105280 | 10.88897071090 |
| N-6 | -84.23483789070 | 10.87701472010 |
| N-7 | -84.21835833300 | 10.86338000000 |
| N-8 | -84.21508833300 | 10.84640666700 |
