# Supplementary material for: Impacts of sediment derived from erosion of partially-constructed road on aquatic organisms in a tropical river: The Río San Juan, Nicaragua and Costa Rica
Source: PLoS One. 2020 Nov 17;15(11):e0242356. doi: 10.1371/journal.pone.0242356 (PMC7671519; doi:10.1371/journal.pone.0242356)
Supplement: S2 Table — The Non-Metric Multidimensional Scaling analysis (NMDS, overall stress = 0.185) showed a segregation of macroinvertebrate assemblages of most sampled deltas across axis 2, showing negative relations with d16, d50 and pH, but positive relations with water temperature, and d84, thereby indicating that macroinvertebrate composition on south bank deltas was influenced by smaller d16, d50, larger d84, and higher water temperature. (DOCX) [file pone.0242356.s002.docx]

**S2 Table. Relations of Vectors of Environmental and Substrate Size Variables with the NMDS axis.**

The Non-Metric Multidimensional Scaling analysis (NMDS, overall stress = 0.185) showed a segregation of macroinvertebrate assemblages of most sampled deltas across axis 2, showing negative relations with d16, d50 and pH, but positive relations with water temperature, and d84, thereby indicating that macroinvertebrate composition on south bank deltas was influenced by smaller d16, d50, larger d84, and higher water temperature.

|  | **NMDS1** | **NMDS2** |
| --- | --- | --- |
| d16 | 0.629155 | -0.77728 |
| d50 | 0.639379 | -0.768892 |
| d84 | 0.71708 | 0.696991 |
| Temperature | 0.277809 | 0.960636 |
| pH | 0.052128 | -0.99864 |
| Conductivity | 0.639044 | -0.76917 |
