# Supplementary material for: Impacts of sediment derived from erosion of partially-constructed road on aquatic organisms in a tropical river: The Río San Juan, Nicaragua and Costa Rica
Source: PLoS One. 2020 Nov 17;15(11):e0242356. doi: 10.1371/journal.pone.0242356 (PMC7671519; doi:10.1371/journal.pone.0242356)
Supplement: S3 Table — Taxa occurring in the Río San Juan identified as sensitive by Carlise et al. (2007) and Zweig and Rabeni (2001). Intermediate (or ‘medium’) sensitivity taxa designated as “ms”; high sensitivity taxa identified as “hs”. (DOCX) [file pone.0242356.s003.docx]

**S3 Table. Sensitivity of Macroinvertebrate Taxa Found in the Río San Juan to Suspended Sediments and Deposited Fine Sediment, Based on Scientific Literature.**

Taxa identified as sensitive by Carlise *et al.* (2007) and Zweig and Rabeni (2001). Intermediate (or ‘medium’) sensitivity taxa designated as “ms”; high sensitivity taxa identified as “hs”.

| **Taxa** | **Suspended Sediments** | **Deposited Fine Sediment** |
| --- | --- | --- |
| **Coleoptera** |  |  |
| **Elmidae** |  | ms(*) |
| **Diptera** |  |  |
| Orthocladiinae |  | hs(+) |
| **Simuliidae** |  |  |
| *Simulium* | ms(*) |  |
| **Tabanidae** | hs(*) |  |
| **Ephemeroptera** |  |  |
| **Caenidae** |  | ms(*), hs(+) |
| *Caenis* | ms(*) |  |
| **Heptageniidae** | ms(*) |  |
| **Leptohyphidae** |  |  |
| *Tricorythodes* |  | hs(+) |
| **Leptophlebiidae** |  | hs(*) |
| **Gastropoda** |  |  |
| **Ancylidae** |  | ms(*) |
| **Heteroptera** |  |  |
| **Veliidae** |  | ms(*) |
| **Odonata** |  |  |
| **Coenagrionidae** |  |  |
| *Argia* |  | hs(+) |
| **Gomphidae** | hs(*) | hs(*) |
| **Plecoptera** |  |  |
| **Perlidae** |  |  |
| *Anacroneuria* | hs(*) | hs(*) |
| **Trichoptera** |  |  |
| **Leptoceridae** | ms(*) | ms(*) |
| *Oecetis* | ms(*) | hs(*) |

(*) Carlise *et al.* 2007

(+) Zweig and Rabeni, 2001

ms= intermediate sensitivity; hs= high sensitivity
